# Multimodal large language model versus emergency physicians for burn assessment: a prospective non-inferiority study

**DOI:** 10.1186/s13049-026-01577-6

**Published:** 2026-02-05

**Authors:** Ahmet Aykut, Ali Rıza Karayıl, Cem Yıldırım, Ertuğ Günsoy, Mehmet Tatlı, Murat Avcı

**Affiliations:** 1 Department of Emergency Medicine, SBU Van Education and Research Hospital, Van, Türkiye; 2 Department of General Surgery, SBU Van Education and Research Hospital, Van, Türkiye

**Keywords:** Burns, Emergency medicine, Large language model, Total body surface area, Diagnostic accuracy

## Abstract

**Background:**

Accurate burn size and depth assessment at first contact guides fluid resuscitation, referral, and operative planning, yet both tasks show meaningful inter-clinician variability. General-purpose multimodal large language models may offer scalable, image-based decision support in emergency care, but prospective benchmarking against clinicians and a robust reference standard remains limited.

**Methods:**

We conducted a prospective, single-centre diagnostic accuracy and agreement study in a tertiary emergency department (22 July–8 September 2025). Consecutive acute burn presentations (< 24 h) were screened; protocol-conformant cases contributed standardized three-view photographs per anatomically distinct burn region. A multimodal large language model generated region-level estimates of total body surface area (TBSA) contribution and burn depth class. Eighteen emergency physicians independently rated the same images and minimal metadata, blinded to model and reference outputs. A three-member expert panel served as the reference standard by consensus. The primary endpoint was non-inferiority of the model versus the physician median for region-level absolute TBSA error relative to the panel, with a pre-specified margin of 3 percentage points, using patient-level cluster bootstrap for inference. Secondary endpoints included TBSA agreement and depth agreement (quadratic-weighted kappa).

**Results:**

Of 413 screened presentations, 52 patients were enrolled, yielding 64 analyzable burn region-cases (35 pediatric, 29 adult). The model’s mean absolute TBSA error versus the panel was 1.40 percentage points (median 1.00); 87.5% of cases were within ± 3 percentage points and 98.4% within ± 5. The physician median had a mean absolute error of 0.89 percentage points (median 0.75). The paired non-inferiority analysis met the pre-specified criterion (Hodges–Lehmann median Δ = 0.25; one-sided 95% upper bound = 0.50), indicating the model was non-inferior to physicians for TBSA estimation. In contrast, depth agreement versus the panel was slight for the model (quadratic-weighted kappa 0.14), with systematic underestimation of deeper burns, while physician consensus showed substantially higher agreement (quadratic-weighted kappa 0.65).

**Conclusions:**

In this prospective emergency department evaluation, a general-purpose multimodal model achieved non-inferior performance to emergency physicians for region-level TBSA estimation but performed substantially worse for burn depth classification. These findings support a narrowly defined adjunct role for TBSA estimation, while depth-dependent decisions should remain clinician-led and require further method development and external validation.

**Supplementary Information:**

The online version contains supplementary material available at 10.1186/s13049-026-01577-6.

## Background

Accurate assessment of burn size and depth is fundamental to early management, guiding fluid resuscitation, referral to specialist centres, operative planning, and outcome prediction. Errors in total body surface area (TBSA) estimation at first contact are common: multiple studies comparing referring hospitals with burn centres have documented systematic overestimation, sometimes by 5–10 percentage points or more, with implications for over-resuscitation, unnecessary transfers, and resource use [1, 2]. Depth assessment is likewise challenging; even experienced clinicians show only moderate agreement for partial-thickness burns when compared with histology or healing-based references [3, 4]. In parallel, smartphone-based telemedicine and image-based consultation have emerged as pragmatic tools to extend burn expertise, generally achieving good concordance for TBSA but more modest performance for depth, especially when based on standard color photographs alone [5, 6]. These limitations motivate the search for scalable, image-based decision-support systems that can stabilize early burn assessment without replacing specialist judgment.

Recent advances in computer vision have produced deep-learning models that segment burn wounds, convert area into %TBSA, and, in some cases, classify depth with performance approaching that of expert raters in curated datasets [7–9]. However, most such systems are tightly coupled to specific training corpora, rarely undergo external validation, and are not yet integrated into routine emergency department workflows. At the same time, mobile health roadmaps emphasize that image-based diagnostic support must be embedded in real-world clinical environments, accounting for front-line constraints, heterogeneous image quality, and organizational factors if it is to improve care [10]. Large Language Models (LLM) now offer a different paradigm: as general-purpose, multimodal systems capable of processing both images and structured clinical metadata, they could, in principle, provide burn assessments without task-specific retraining, and could be updated over time as the underlying models evolve.

Within emergency medicine, LLMs are being explored for triage, diagnostic reasoning, and documentation, but most evaluations to date have used vignettes or retrospective test sets, with limited prospective assessment of performance against clinician benchmarks in real patients [11, 12]. To our knowledge, no prior study has prospectively compared a general-purpose multimodal LLM with emergency physicians for region-level burn assessment using standardized clinical photographs and a multi-expert reference standard. We therefore conducted a prospective, single-centre diagnostic accuracy and agreement study in a tertiary emergency department, comparing LLM-based TBSA and depth estimates with those of emergency physicians, using a three-member expert panel as the reference. The primary objective was to evaluate whether the LLM’s TBSA estimates were not meaningfully worse than those of emergency physicians, and secondary objectives included comparing depth agreement, calibration, and subgroup performance by age and anatomical region.

## Methods

### Study design and setting

This prospective, observational, single-centre diagnostic accuracy and agreement study was conducted in the emergency department (ED) of a tertiary academic hospital. The accrual window was 22 July 2025 to 8 September 2025, during which all consecutive ED presentations for acute burns were screened. The work compared region-level burn-extent (TBSA) and burn-depth estimations produced by a LLM and by emergency physicians against a three-member expert-panel reference standard. An institutional non-interventional protocol prespecified the setting, eligibility, and core analyses; procedures followed good clinical practice and the Declaration of Helsinki with written consent for clinical imaging.

### Participants and unit of analysis

We enrolled consecutive emergency department presentations for acute burns, defined as the first presentation within 24 h of injury. No exclusions were made based on burn mechanism (scald, flame, contact, chemical, electrical, or mixed). We did not record the exact time since injury beyond the < 24-h threshold.

The analytic unit was the burn region. For multi-region injuries, each region photographed per protocol constituted a separate region-case and was analyzed independently. Region-level TBSA represents the percentage-point contribution of that region to the patient’s total body surface area, counting only partial-thickness or deeper burns and excluding purely epidermal/erythema-only areas. Screening and enrolment followed prospective consecutive sampling during routine ED operations; imaging was performed after initial stabilization and before extensive dressing, whenever feasible. Adults provided written consent; legal guardians consented for minors, with assent obtained when appropriate.

Exclusions were: (i) no consent/assent; (ii) failure to obtain protocol-conformant images (three standardized views with adequate quality); (iii) follow-up or chronic-wound visits for the same episode; (iv) non-burn injuries or isolated inhalation injury without visible cutaneous burns; and (v) situations in which imaging was not clinically safe.

Regions were defined a priori using age-appropriate Lund–Browder anatomical areas. If a burn extended contiguously across multiple Lund–Browder areas, each anatomically distinct area photographed per protocol was treated as a separate region-case, and region-level TBSA reflected only that area’s percentage-point contribution. The decision to exclude erythema-only (epidermal) burns and to enrol only injuries with suspected partial-thickness or deeper involvement was made at the bedside before photography by the burn ED clinician responsible for initial assessment.

### Index test and comparator

The index test was a single multimodal large language model (gpt-5-chat-latest; OpenAI, San Francisco, CA, USA) accessed via the OpenAI HTTPS API. All inferences for this study were run on 30 December 2025 via the public “gpt-5-chat-latest” endpoint on the OpenAI platform; no other models or vendor endpoints were used. At the time of the study, this endpoint was documented by the provider as a general-purpose, instruction-following model capable of joint text–image reasoning. Browser tools and external web access were disabled. For each region-case, we submitted exactly one API request containing three standardized photographs (one orthogonal anterior and two oblique views) together with minimal clinical metadata (age, sex, height, weight, and the target anatomical region). The frozen prompt (Appendix A) specified a strict JSON schema with three required keys: (i) tbsa_percent (0–100, percentage points contributed by the imaged region), (ii) depth_class (categorical: superficial_partial, deep_partial, or full_thickness), and (iii) rationale (free text, ≤ 80 words). The prompt was finalized before running any study cases and was not iteratively tuned on study images; after freezing, no informal prompt adjustments were made and there was no case-wise prompt modification. Sampling was configured to be deterministic (temperature 0.0, top_p 1.0, one completion per case); no manual editing, re-running, or case-wise prompt modification was performed. Images were resized, when necessary, so that the long edge did not exceed 1400 pixels, recompressed at JPEG quality 80, and stripped of EXIF metadata before transmission. The API timeout was set to 90 s as a pragmatic upper bound to avoid indefinite waits; if a request failed for technical reasons (e.g. transport or server error), it was automatically retried once with identical parameters. The resulting JSON outputs were stored verbatim, and the final analysis dataset was frozen with cryptographic checksums and a methods manifest. The free-text “rationale” field was retained verbatim for transparency/auditability and was reviewed qualitatively for plausibility and to inform error characterization, but it was not treated as an endpoint and was not used in any quantitative inferential analyses.

Emergency physicians served as the comparator group. Eighteen board-certified or board-eligible emergency physicians independently rated each region-case using the same three photographs and minimal metadata provided to the LLM. Raters were blinded to the LLM and reference-standard outputs and to each other; case order was individually randomized. Assessments were captured in a secure electronic case-report form with mandatory fields for TBSA estimate, depth class (using the same three-category ontology), and image-quality flags. Before scoring, each rater received a brief calibration packet including two exemplar cases per depth class and a refresher on age-appropriate Lund–Browder charts.

All case-level LLM outputs (tbsa_percent, depth_class, and free-text rationale), together with the prompt-level age and anthropometry text, are provided in de-identified form in Supplementary Table S1.

### Reference standard (expert panel)

A three-member expert panel (one burn surgeon, two emergency physicians) served as the reference standard. Panelists independently estimated region-level TBSA using age-appropriate Lund–Browder charts and classified depth (superficial partial, deep partial, full thickness) from the same de-identified images and minimal metadata. Non-assessable was allowed when SOP failures precluded valid interpretation. Discrepancies triggered a structured adjudication: automatic acceptance when TBSA range ≤ 2 percentage-points and majority depth agreement; otherwise, an anonymized round-robin re-score followed by a brief synchronous review as needed. Final consensus used the median TBSA and majority depth (three-way ties defaulted to deep partial absent unanimity). Pre-consensus agreement was summarized by ICC (TBSA) and quadratic-weighted kappa (depth).

### Outcomes

Primary endpoint: Region-level absolute error in TBSA (%) for the LLM versus the expert-panel consensus, tested for non-inferiority against the physician median absolute error. The non-inferiority margin (Δ*) was 3 percentage-points a priori. We prespecified a non-inferiority margin (Δ) of 3 percentage points to represent clinical equivalence for routine ED decision-making rather than burn-center–level precision. This choice reflects that early ED management and referral decisions are typically guided by pragmatic TBSA cut-points and that small absolute differences in regional TBSA estimates within the low single-digit range are unlikely to alter initial fluid resuscitation and disposition decisions in routine practice. In addition, prior literature documents non-trivial inter-clinician variability in TBSA estimation even when standardized charts are used, supporting a low single-digit tolerance as a reasonable “no worse than typical practice” margin for a non-inferiority framework [13, 14].

Key secondary endpoints: (i) Depth agreement (quadratic-weighted kappa) for LLM and for individual physicians versus reference; (ii) continuous agreement for TBSA via Lin’s concordance correlation coefficient (CCC) and Bland–Altman bias/limits; (iii) threshold-based clinical accuracy: adults ≥ 20% and pediatrics ≥ 10–15% TBSA (sensitivity, specificity, ROC–AUC; DeLong comparisons versus physician-median); (iv) inter-rater variability among physicians (two-way random-effects ICC; dispersion of absolute errors); (v) calibration (slope/intercept and bin-wise calibration error); (vi) sensitivity excluding region-cases with major image-quality flags.

### Sample size and power

This study was conceived as a pragmatic prospective diagnostic accuracy and agreement evaluation embedded in routine ED operations, with a fixed accrual window rather than a formal a priori power calculation. Over the accrual window we obtained 64 analyzable region-cases. Secondary and subgroup analyses were not powered a priori and should be interpreted cautiously.

### Statistical analysis

#### Populations

The Full Analysis Set included region-cases with protocol-conformant imaging and valid outputs from the LLM, at least one physician, and the reference panel. A Per-Protocol set excluded region-cases rated non-assessable by ≥ 2 panelists. For patients with burns involving multiple anatomically distinct regions, each photographed region was treated as a separate region-case; consequently, some patients contributed more than one observation, inducing clustering at the patient level.

#### Descriptives

Continuous variables were summarized as mean ± SD or median [IQR]; categorical variables as n(%). TBSA values represent percentage-point contributions of the region to total body area.

#### Primary analysis

Let Δ_i be the paired difference between LLM absolute error and the physician-median absolute error for region-case i. Non-inferiority was declared if the upper bound of the one-sided 95% CI for the Hodges–Lehmann median of Δ_i (cluster bootstrap, 5,000 resamples clustered by patient) was ≤ Δ*; superiority if the upper bound < 0. All primary and secondary inferential analyses accounted for within-patient clustering using patient-level cluster bootstrap resampling (patients sampled with replacement; all regions retained within each replicate); for agreement metrics (e.g., κ, CCC), cluster-bootstrap confidence intervals were computed using 2,000 replicates. No mixed-effects models were fitted for the primary or secondary endpoints; inference relied on the prespecified patient-level cluster bootstrap framework.

#### Secondary analyses

Depth agreement by quadratic-weighted kappa; continuous agreement by CCC and Bland–Altman; threshold accuracy by ROC–AUC with DeLong comparisons; physician inter-rater variability by ICC; calibration via regression/plots; image-quality sensitivity. Exploratory subgroup analyses (age < 18 vs ≥ 18; anatomical region) were descriptive and used the same patient-level cluster bootstrap approach within strata; no mixed-effects interaction models were fitted. Missing data were not imputed for primary/secondary outcomes; values outside [0,100] were flagged as entry errors.

#### Image acquisition and quality assurance

Photographs were obtained prior to debridement and before application of any agents that could alter wound appearance (e.g., colored antiseptics); when needed, only gentle rinsing with clear saline/water was used to remove gross debris without changing skin coloration. Images were captured using available smartphone cameras during routine ED care, while meeting the prespecified minimum image-quality requirements described below.. Per region-case we captured three standardized views (one orthogonal anterior, two oblique ≈ ± 30–45°). Minimum specifications: long-edge ≥ 2,000 px; native capture without digital zoom; adequate focus and diffuse illumination; a metric ruler or 1-cm grid in frame when feasible (palmar surface acceptable with documentation); JPEG quality ≥ 90% or lossless; framing included the entire lesion plus 2–3 cm of surrounding skin. Images failing critical criteria (resolution/focus/exposure) were excluded for that region with documented reason codes. Faces/identifiers were masked; EXIF data were stripped; files were stored in encrypted repositories with access logs.

#### Ethics and reporting

The institutional non‑interventional protocol and local approvals governed conduct. Written consent for clinical photography was obtained from adults and from legal guardians for minors. Reporting follows STROBE and STARD for observational and diagnostic-accuracy elements, and was cross-checked against the STARD-AI extension for AI-centred diagnostic accuracy studies; AI-specific reporting adheres to CLAIM [15, 16]. The frozen prompt (Appendix A) and model settings are archived.

## Results

**Fig. 1:**
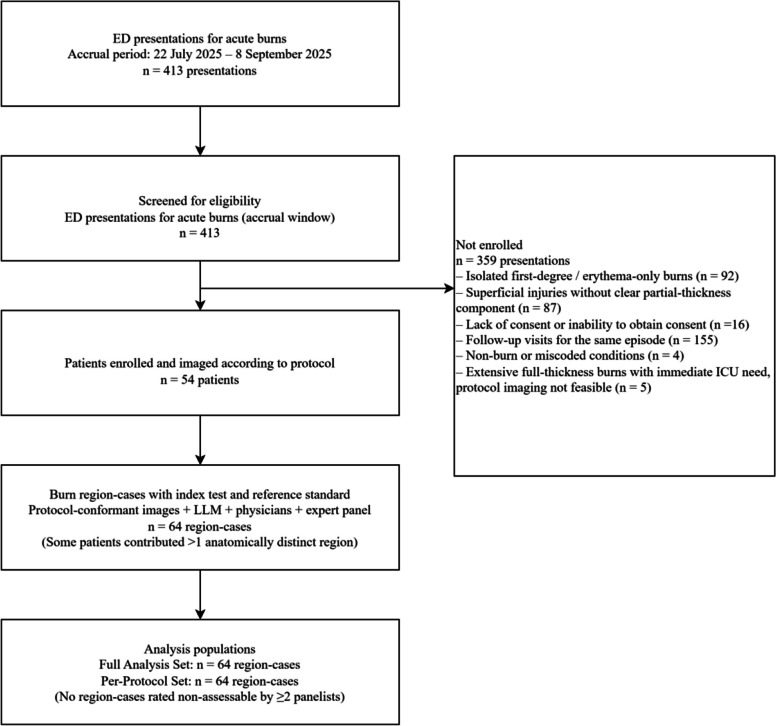
Flow of emergency department burn presentations, enrolled patients, and analysed burn region-cases. During the accrual period, 413 presentations were screened, 52 patients were enrolled, and 64 region-cases were included in both the Full Analysis and Per-Protocol Sets

During the accrual period (22 July 2025 to 8 September 2025), 413 emergency department presentations for acute burns were screened. Of these, 52 patients were enrolled and imaged according to the study protocol, contributing a total of 64 burn region-cases because some patients had burns in more than one anatomically distinct region. The remaining 359 presentations were not enrolled for reasons including isolated first-degree/erythema-only burns outside the study scope, superficial injuries without clear partial-thickness involvement, lack of consent or inability to obtain consent, follow-up visits for the same episode, and non-burn or miscoded conditions (with counts for each reason shown in Fig. 1). Five additional presentations involved extensive full-thickness burns with immediate ICU need in which protocol-conformant photography was not clinically feasible (Fig. 1).

Among the 52 unique patients, 43 contributed one region-case, six contributed two region-cases, and three contributed three region-cases. Of these 64 region-cases, 35 occurred in patients younger than 18 years and 29 in adults aged 18 years or older. All 64 region-cases had complete TBSA and depth outputs from the LLM, the physician group, and the expert panel and were therefore included in the Full Analysis Set and the Per-Protocol set. No region-cases were excluded due to non-assessability by the expert panel. Baseline characteristics of the region-cases, including age distribution, anatomical sites, panel depth classes, and image-quality scores, are summarized in Table 1. The expert panel demonstrated excellent inter-rater agreement, with ICC = 0.97 for TBSA estimation and quadratic-weighted κ = 0.86 for depth classification, supporting the use of the panel consensus as the reference standard.

**Table 1:** Characteristics of included region-cases

Characteristic	Overall (N = 64)
*Number of region-cases*	
Age < 18 years, n (%)	35 (54.7%)
Age ≥ 18 years, n (%)	29 (45.3%)
Age, years, median [IQR]	12.5 [2.8–32.5]
Age < 18 years, median [IQR]	3.0 [1.2–6.0]
Age ≥ 18 years, median [IQR]	35.0 [24.0–42.0]
Image quality (1–10), median [IQR]	9.0 [8.0–9.0]
*Anatomical region*	
Face/head	7 (10.9%)
Torso	6 (9.4%)
Upper extremity	21 (32.8%)
Lower extremity	30 (46.9%)
*Panel depth*	
Superficial partial	47 (73.4%)
Deep partial	16 (25.0%)
Full thickness	1 (1.6%)

Values are n (%) or median [interquartile range]. Image quality was rated on a 1–10 scale by emergency physician raters. Each row describes one burn region (“region-case”) in the Full Analysis Set

*IQR* interquartile range

**Fig. 2:**
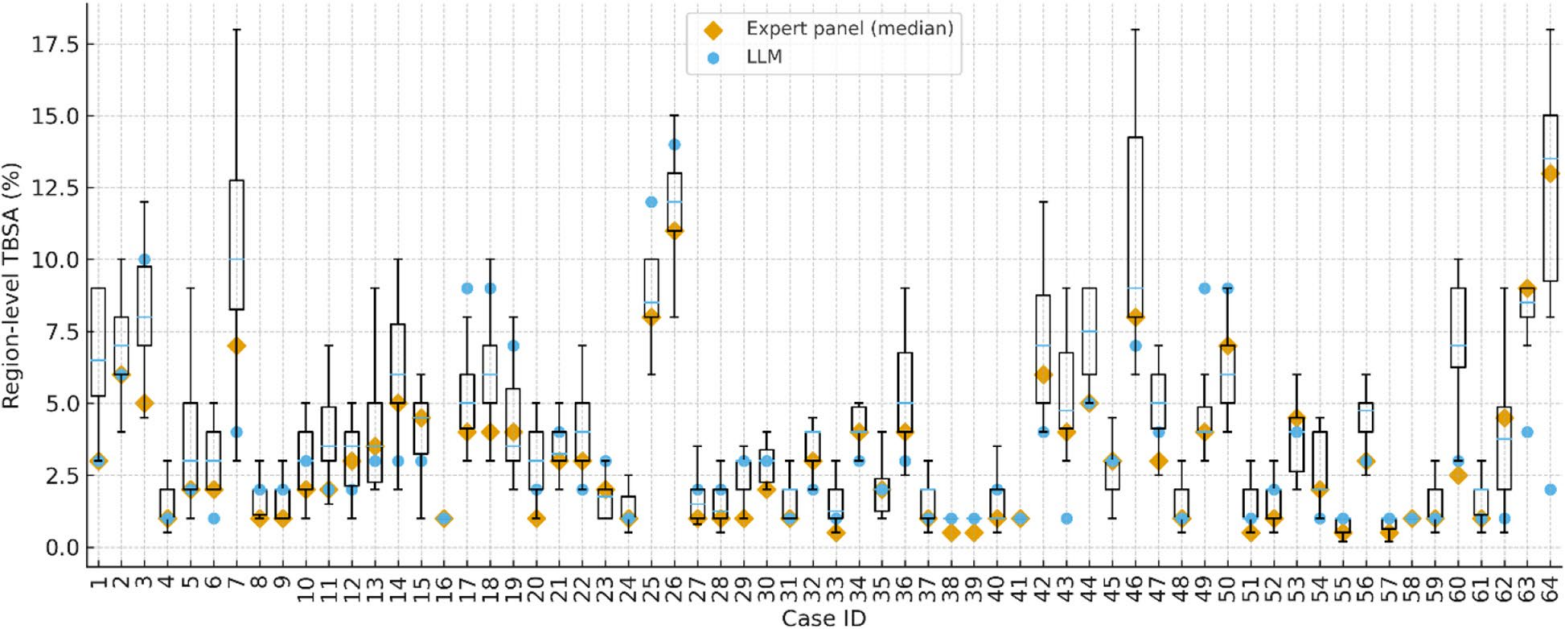
Case-wise region-level burn size estimates for 64 acute burn regions. For each case on the x-axis, the white boxplots depict the distribution of TBSA assessments from 18 emergency physicians (median, interquartile range, and whiskers). The boxplots summarize physician-to-physician variability for each case and are not a separate marker series linked to the orange (panel) or blue (LLM) point estimates. Orange diamond markers indicate the expert panel median TBSA for that case, and blue circular markers represent the TBSA estimated by the large language model (LLM). The y-axis shows region-level TBSA in percentage points

When compared with the expert panel, the LLM showed small errors in estimating TBSA. The mean absolute error between LLM and panel TBSA was 1.40 percentage points, and the median absolute error was 1.00 percentage point. Overall, 87.5% of cases were within ± 3 percentage points of the panel TBSA and 98.4% were within ± 5 percentage points. Continuous agreement, quantified by Lin’s CCC, was 0.642, indicating moderate concordance between LLM and panel TBSA values (cluster-bootstrap 95% CI 0.347 to 0.851). Bland–Altman analysis of the difference between LLM and panel TBSA (LLM minus panel) demonstrated a mean bias of + 0.15 percentage points, with 95% limits of agreement ranging from − 4.47 to + 4.76 percentage points, suggesting that most LLM estimates lay within a narrow and clinically acceptable range around the expert reference. These summary accuracy and agreement metrics are presented in Table 2, and the case-wise distributions of panel, LLM, and physician TBSA estimates are illustrated in Fig. 2.

**Table 2:** Region-level TBSA accuracy and agreement with the expert panel

Method	LLM vs Panel	Physician median vs Panel
Mean absolute error (p.p.)	1.4	0.89
Median absolute error (p.p.)	1	0.75
Within ± 3 p.p., n (%)	56 (87.5%)	62 (96.9%)
Within ± 5 p.p., n (%)	63 (98.4%)	64 (100.0%)
Lin’s CCC	0.642	0.897
Mean bias (p.p.)	0.15	0.78
95% LoA (p.p.)	− 4.47 to 4.76	− 1.20 to 2.76

Absolute error is defined as the absolute difference (in percentage points) between the method and the expert panel TBSA for each burn region. “Within ± 3 p.p.” and “Within ± 5 p.p.” denote the proportion of regions with absolute error ≤ 3 and ≤ 5 percentage points, respectively

*TBSA* total body surface area, *LLM* large language model, *p.p.* percentage points, *CCC* Lin’s concordance correlation coefficient, *LoA* limits of agreement

**Fig. 3:**
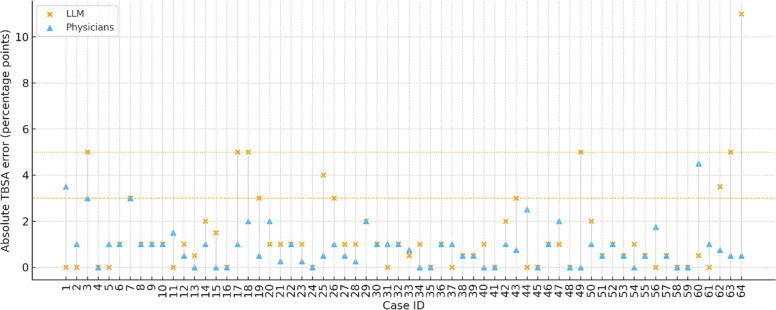
Absolute TBSA error of the LLM and emergency physicians relative to the expert panel across 64 burn regions. For each case on the x-axis, the vertical pair of markers shows the absolute error (in percentage points on the y-axis) of the LLM (orange “ × ” marker) and the physician median (blue triangular marker). Horizontal dashed reference lines at 3 and 5 percentage points indicate the pre-specified non-inferiority margin and the wider ± 5-percentage-point error band, respectively

The pre-specified non-inferiority analysis compared LLM absolute error with the median absolute error of emergency physicians for each region-case, both referenced to the panel. The distribution of the paired differences Δ_i (LLM absolute error minus physician-median absolute error) was centered at zero (Hodges–Lehmann median 0.25 percentage points), and the one-sided 95% confidence interval (CI) for this median had an upper bound of 0.50 percentage points. This upper bound was well below the non-inferiority margin of 3 percentage points and did not meet the criterion for superiority (upper bound < 0), indicating that the LLM was non-inferior to the physician consensus with respect to region-level TBSA error, with no clear evidence of systematic advantage or disadvantage. In a sensitivity analysis restricted to one randomly selected region-case per patient, the non-inferiority conclusion was unchanged (the one-sided 95% upper bound remained < 3 across repeated random selections). The paired absolute errors for each case and the non-inferiority thresholds are displayed in Fig. 3. No supportive mixed-effects model was fitted for this endpoint; the prespecified patient-level cluster bootstrap analysis constituted the inferential comparison for the primary outcome.

Emergency physicians showed somewhat smaller TBSA errors than the LLM when directly compared with the panel. The mean absolute error between the physician median and the panel TBSA was 0.89 percentage points (median 0.75 percentage points), and 96.9% and 100% of cases were within ± 3 and ± 5 percentage points of the panel, respectively. Concordance between physician median and panel TBSA was high (Lin’s CCC 0.897; cluster-bootstrap 95% CI 0.773 to 0.954). Bland–Altman analysis for physicians demonstrated a mean bias of + 0.78 percentage points, with 95% limits of agreement from − 1.20 to + 2.76 percentage points, indicating that physicians tended to slightly overestimate TBSA relative to the expert panel but with a narrower dispersion than the LLM. Across the 18 emergency physicians, inter-rater variability for TBSA was modest: the intra-class correlation coefficient (two-way random effects, absolute agreement) was 0.71, indicating substantial agreement in TBSA estimation among physicians (Table 2).

Calibration analyses further illustrated these differences. For the LLM, regressing LLM TBSA on panel TBSA yielded an intercept of 1.05 and a slope of 0.71 (R^2^ = 0.42), suggesting some compression of extremes with a tendency to pull values toward the center of the distribution. For the physician median, the corresponding intercept and slope were 0.74 and 1.01 (R^2^ = 0.88), indicating near-identity calibration relative to the panel with substantially higher explained variance. In a complementary comparison using the physicians’ median TBSA as the reference, the LLM again demonstrated similar performance: the mean absolute error between LLM and physician median TBSA was 1.57 percentage points, and the median absolute error was 1.00 percentage point. In this comparison, 84.4% of cases were within ± 3 percentage points and 96.9% within ± 5 percentage points of the physician consensus. Lin’s CCC for LLM versus physician consensus TBSA was 0.63 (cluster-bootstrap 95% CI 0.361 to 0.834), closely mirroring the panel-referenced estimate and reinforcing that the LLM’s TBSA estimates were tightly clustered around both expert and clinician assessments.

Agreement on burn depth classification was lower than for TBSA. Using the three-level ontology, the expert panel classified 47 of 64 cases (73.4%) as superficial partial, 16 cases (25.0%) as deep partial and 1 case (1.6%) as full thickness. The LLM, in contrast, assigned 59 cases (92.2%) to superficial partial and 5 cases (7.8%) to deep partial, and did not label any case as full thickness. Exact agreement between the LLM and the panel on burn depth was observed in 48 of 64 cases (75.0%). However, most disagreements reflected underestimation of depth: 13 of the 16 panel-rated deep partial cases were classified as superficial partial by the LLM, and the single full thickness case was also downgraded to superficial partial. The resulting quadratic weighted kappa for burn depth between the LLM and the expert panel was 0.14 (95% CI − 0.06 to 0.39), indicating only slight agreement beyond chance despite the relatively high proportion of exact matches and highlighting that most discrepancies occurred at clinically relevant category boundaries. In practical terms, κ = 0.14 reflects near-chance agreement beyond chance correction. The discrepancy between a relatively high raw agreement (75%) and a low κ is consistent with a prevalence/marginal-imbalance (“kappa paradox”) effect, whereby dominant use of one category (here, superficial partial) and sparse use of others (notably full thickness) inflates chance agreement and depresses κ. Notably, the LLM did not correctly identify the single full-thickness case and systematically downgraded deeper burns (deep partial and full thickness) to superficial partial, a pattern that carries clinical risk for depth-dependent decisions. Depth distributions and agreement metrics for all assessor groups are summarized in Table 3, and full confusion matrices for the three-level ontology are provided in Supplementary Table S2A–C.

**Table 3:** Burn depth distributions and agreement metrics across assessors

Measure	Panel	LLM	Physician consensus
Superficial partial, n (%)	47 (73.4%)	59 (92.2%)	42 (65.6%)
Deep partial, n (%)	16 (25.0%)	5 (7.8%)	19 (29.7%)
Full thickness, n (%)	1 (1.6%)	0 (0.0%)	3 (4.7%)
Exact agreement with panel, n/N (%)	–	48/64 (75.0%)	54/64 (84.4%)
Quadratic weighted kappa vs panel	–	0.144	0.648
Exact agreement with physician consensus, n/N (%)	54/64 (84.4%)	42/64 (65.6%)	–
Quadratic weighted kappa vs physician consensus	0.648	0.128	–

Depth categories are based on a three-level ontology (superficial partial, deep partial, full thickness). “Exact agreement with panel/physician consensus” denotes the number and percentage of regions for which the depth class was identical to the reference. Quadratic weighted kappa quantifies agreement on the three-level depth scale. Panel refers to the three-member expert panel; physician consensus to the modal depth class across 18 emergency physicians

*LLM* large language model

Emergency physicians showed substantially higher agreement with the expert panel for burn depth. Based on the modal class across the 18 physicians, superficial partial, deep partial and full thickness were assigned in 42 (65.6%), 19 (29.7%) and 3 (4.7%) cases, respectively. Overall, 54 of 64 cases (84.4%) were classified identically by the physician consensus and the panel, and most discrepancies occurred between adjacent categories. The quadratic weighted kappa for physician consensus versus the panel was 0.69 (95% CI 0.44 to 0.89), consistent with substantial agreement, whereas the κ for LLM versus physician consensus was 0.13 (95% CI − 0.06 to 0.34), again in the slight-agreement range observed for the LLM–panel comparison (Supplementary Table S2A–C). When the LLM was compared directly with the physicians’ modal depth classification, exact agreement was present in 42 of 64 cases (65.6%). Together, these findings suggest that, in this setting, the LLM reproduced panel-level TBSA estimates with small numerical error but tended to systematically underestimate burn depth relative to both experts and frontline clinicians, particularly for deeper injuries (Table 3).

Exploratory subgroup analyses did not identify strong effect modification by age or anatomical region. In pediatric cases, the mean absolute error in TBSA was 1.39 percentage points for the LLM and 0.96 percentage points for the physician median, with medians of 1.0 percentage point for both methods. In adult cases, the corresponding mean absolute errors were 1.41 and 0.80 percentage points, with medians of 0.5 percentage points. Region-stratified summaries showed slightly larger LLM errors for torso and face burns than for upper and lower extremity burns, a pattern that was also observed, albeit to a lesser extent, for physician estimates. However, the small number of cases per region limited the precision of subgroup comparisons, and no consistent pattern of systematic over- or under-performance of the LLM in any particular subgroup was evident.

Pre-specified sensitivity analyses based on image quality were constrained by the uniformly high quality of the photographic inputs. The median physician-rated image quality was 9 on the 1–10 scale (interquartile range 8–9), and only a small minority of ratings fell below 8 (Table 1). Within this narrow range, there was no meaningful association between image quality and absolute TBSA error for either the LLM or the physicians (Spearman ρ ≈ 0.13 for the LLM and ρ ≈ 0.02 for physicians, both p > 0.3), and excluding the few lower-rated cases did not materially change any of the primary or secondary estimates. Threshold-based discrimination analyses for larger regional TBSA contributions were limited by the small number of high-TBSA regions in this sample and did not reveal major qualitative differences between the LLM and physician consensus.

## Discussion

In this prospective, single-centre evaluation, a LLM achieved non-inferior performance to emergency physicians for estimating burn size, while performing distinctly worse for burn depth classification. The model’s region-level TBSA estimates were generally close to the expert-panel reference and to clinicians’ assessments, remaining within the pre-specified non-inferiority margin selected for clinical relevance. By contrast, agreement with experts for depth was only slight, whereas emergency physicians achieved substantially higher concordance. These findings support a narrow adjunct role for LLM-based tools in TBSA estimation under standardized photographic conditions, but indicate that depth-dependent decisions should remain under expert clinical control and, where available, be supported by dedicated modalities.

Our TBSA results align with the established literature showing variability in human burn assessment. A systematic review synthesizing 28 studies reported that TBSA estimates often diverge between referring clinicians and burn centres, with a tendency toward overestimation early in care [3]. Swords et al. similarly found substantial overestimation at referring institutions for transferred children, and linked greater overestimation to clinically important fluid over-resuscitation [17]. In contrast, embedded burn expertise appears to improve agreement: Yoo et al. reported moderate-to-substantial agreement and excellent reliability between emergency physicians and burn-unit staff, with mean differences in exact TBSA estimates in the low single digits [14]. Together, these data support the premise of our non-inferiority design—that low single-digit TBSA discrepancies reflect routine ED practice rather than burn-centre precision—and contextualize our finding that the LLM’s TBSA accuracy was comparable to emergency physicians in this setting.

Depth assessment remains a more error-prone task. Even experienced clinicians can misclassify depth compared with histology or healing-based reference standards [3, 5, 18, 19]. Telemedicine and smartphone-based burn evaluation studies similarly show good TBSA estimation but more modest depth performance, with frequent misclassification around partial-thickness boundaries [5, 18, 20]. In our study, the LLM’s depth agreement was only slight despite a superficially high raw agreement, consistent with a prevalence/marginal-imbalance (“kappa paradox”) effect when one category dominates and sparse categories contribute disproportionately to chance agreement. Critically, the LLM did not identify the full-thickness case and systematically downgraded deeper injuries, a pattern that creates clinical risk for depth-dependent management.

Recent computer-vision work provides useful context. Dedicated deep-learning systems trained on curated burn-image datasets have reported strong performance for segmentation and TBSA estimation [7, 8], and some hybrid or convolutional approaches have reported high accuracy/AUC for predicting severity or surgical need [7, 21, 22]. However, a systematic review highlighted frequent limitations including small homogeneous datasets, bias, and limited external validation—particularly for skin-tone diversity and real-world acquisition variability [21]. elemedicine studies likewise show that while remote assessment can improve access, depth classification often remains less reliable and may require adjunctive technologies such as laser Doppler imaging [5, 18, 19]. Against this background, our prospective ED dataset and expert-consensus reference demonstrate that a general-purpose LLM can approximate clinician TBSA performance, yet does not approach the depth-classification performance reported for task-optimized imaging systems—underscoring that depth grading likely requires modality- and task-specific optimization rather than general-purpose multimodal reasoning.

More broadly, our results echo themes in the emerging emergency-medicine LLM literature: promising stand-alone capability does not automatically translate into dependable clinical utility. Reviews emphasize that most evidence remains based on vignettes or bench testing rather than prospective clinical evaluations [12, 23, 24]. In a randomized trial of GPT-4 as a diagnostic aid, providing model access did not significantly improve physicians’ diagnostic reasoning compared with conventional resources [25]. Similarly here, the LLM performed well on a constrained geometric task (TBSA estimation) yet failed on a closely related but clinically consequential task (depth), suggesting that deployment should focus on tightly specified roles with explicit escalation pathways rather than broad automation expectations.

This study has strengths that support interpretability: prospective consecutive enrolment, standardized photography in a real ED, an adjudicated multi-expert reference standard, and blinded assessments by 18 emergency physicians using the same inputs as the LLM. The TBSA evaluation was anchored in a pre-specified non-inferiority framework with a clinically motivated margin [3, 14, 17]. However, key limitations constrain generalizability. This was a single academic centre with co-located burn expertise; external validity to community hospitals, pre-hospital care, or low-resource settings is uncertain. Although TBSA estimation is often less accurate in non-specialist facilities [3, 5, 17]**,** LLM performance under such conditions should not be assumed to improve. Image quality in our dataset was uniformly high and protocolized; performance may degrade with lower resolution, motion blur, occlusion, suboptimal framing, or non-standard lighting. Skin-tone diversity and illumination variability were limited, and generalization across pigmentation and lighting conditions remains uncertain. We evaluated one model configuration at one time point; performance may change with version updates or hybrid architectures. Finally, we did not compare against state-of-the-art computer-vision burn tools or assess downstream effects on clinician behavior or patient outcomes. Accordingly, our results should be interpreted as a bounded snapshot of general-purpose multimodal LLM capabilities in ED burn assessment rather than a ceiling estimate of AI performance.

In practical terms, our findings support consideration of an LLM as a second reader for TBSA estimation under standardized imaging workflows, while treating depth outputs as unreliable and non-actionable. Depth-dependent management should remain clinician-led and, where feasible, supported by adjunctive technologies such as laser Doppler imaging [19]. Future work should prioritize multi-centre external validation, more diverse imaging and skin-tone distributions, and evaluation of human–AI collaboration within real workflows to determine whether TBSA-support tools can safely improve consistency and equity in acute burn care [5, 10, 18, 20].

## Conclusion

In this prospective, image-based diagnostic study, a general-purpose multimodal LLM achieved non-inferior performance to emergency physicians for estimating region-level TBSA, with small absolute errors and minimal bias relative to a multi-expert panel, suggesting potential utility as an adjunct for early burn size quantification in the emergency department, particularly where specialist burn expertise is limited. However, the model systematically underestimated burn depth and showed only slight agreement with expert and clinician depth classifications, indicating that it is not yet suitable for decisions that hinge on accurately distinguishing deep partial- from full-thickness burns. These findings highlight both the promise and current limitations of LLM-based tools in acute burn care: they may help stabilize early fluid resuscitation and referral decisions by improving TBSA estimation, but safe clinical integration will require further development and validation of depth assessment and careful oversight of model use in frontline workflows.

## Supplementary Information

Supplementary Material 1.

Supplementary Material 2.

Supplementary Material 3.

## Data Availability

The anonymised analysis dataset and the statistical code (R scripts) used in this study will be made available in a public repository upon acceptance of the manuscript. Until then, de-identified data and analysis code are available from the corresponding author on reasonable request, subject to institutional and data protection regulations.

## Abbreviations

TBSA: Total body surface area
LLM: Large language model
ED: Emergency department
