# Supplementary material for: Multimodal large language model versus emergency physicians for burn assessment: a prospective non-inferiority study
Source: Scand J Trauma Resusc Emerg Med. 2026 Feb 5;34:54. doi: 10.1186/s13049-026-01577-6 (PMC12969848; doi:10.1186/s13049-026-01577-6)
Supplement: Supplementary file 2 — Supplementary Material 2. [file 13049_2026_1577_MOESM2_ESM.docx]

| **case_no** | **age_text** | **height_cm** | **weight_kg** | **region** | **tbsa_percent** | **depth_class** | **rationale** |
| --- | --- | --- | --- | --- | --- | --- | --- |
| 1 | Age: 1.5 years | 60 | 12 | Right upper extremity | 3 | superficial_partial | Right arm shows patchy, moist, bright-pink areas with thin-walled blisters and skin slough, indicating superficial partial-thickness. No leathery eschar or pale, insensate zones. Burn area roughly two to three child palms; erythema-only zones excluded. This approximates ~3% TBSA contribution from the right upper extremity. |
| 2 | Age: 1.5 years | 60 | 12 | Right lower extremity | 6 | superficial_partial | Moist, bright red burn with epidermal peeling/blister remnants over posterior–lateral thigh, crossing the knee to a small proximal leg area; no waxy white, mottled, or leathery eschar. These cues indicate superficial partial thickness. Coverage is ~40% of the right lower limb (thigh mostly involved, small proximal leg patch; foot largely spared). A toddler leg ≈14% TBSA → ~0.4 of leg ≈ 6% TBSA. |
| 3 | Age: 1.5 years | 60 | 12 | Thorax | 10 | superficial_partial | The thoracic area shows moist, pink, shiny burns with intact pain response and some blistering, indicating superficial partial-thickness injury. No leathery or pale insensate zones are noted. In a 1.5-year-old, the anterior thorax represents about 10% TBSA, which aligns with the visibly affected area excluding erythema-only regions. |
| 4 | Age: 10 months | 50 | 10 | Right hand | 1 | superficial_partial | Dorsal right hand and dorsal aspects of three fingers show moist pink-red dermis with deroofed and intact blisters and weeping. No leathery/charred eschar or dry, white, insensate areas are seen. These cues indicate superficial-partial thickness. The involved area is roughly the size of one infant palm (including fingers), about 1% of total body surface. |
| 5 | Age: 4 years | 100 | 14 | Face | 2 | superficial_partial | Moist pink dermis with epidermal loss and ruptured blisters over nose, upper lip, and medial cheeks; visible weeping/bleeding suggests preserved capillary refill. No waxy white, mottled, or leathery eschar to suggest deeper injury. Affects central face only, about 1.5–2 child palms in area (~2% TBSA). |
| 6 | Age: 1 years | 60 | 9 | Right upper extremity | 1 | superficial_partial | Burn limited to a narrow band of the distal forearm/wrist with a small patch onto the hand; overall area approximates one child palm (~1% TBSA). The wound is pink-red and moist/shiny with superficial epidermal loss; no leathery/charred eschar or mottled white areas. Hair follicles/skin lines remain visible—consistent with superficial partial-thickness injury. |
| 7 | Age: 8 months | 60 | 8 | Left lower extremity | 4 | superficial_partial | Moist bright‑pink denuded skin with intact/ruptured blisters on the anterior distal leg, dorsum of foot, and a small anterior knee patch. No leathery eschar or waxy pallor; color suggests brisk capillary refill—consistent with superficial partial thickness. Involvement ≈ distal two‑thirds of anterior/lateral leg plus most dorsal foot (~30% of one leg). One infant leg ≈14% TBSA → ~4% (excluding surrounding erythema). |
| 8 | Age: 2 years | 81 | 11 | Right lower extremity | 2 | superficial_partial | Pink, moist dermis with epidermal peeling and small clear blisters across the dorsum/ankle and some plantar toes. No waxy white areas, eschar, or leathery texture. Appearance suggests brisk capillary refill. These cues indicate superficial partial-thickness injury. Involved area approximates about two child handprints (most of foot with circumferential ankle, calf spared), ≈2% TBSA. |
| 9 | Age: 10 years | 150 | 45 | Right lower extremity | 2 | superficial_partial | Clear, tense blisters on toes and pink, moist dermis on the medial ankle after epidermal loss indicate superficial partial-thickness. No leathery/white eschar, charring, or dry insensate areas. Involvement limited to toes/dorsal foot plus a medial ankle patch, totaling about 1–2 palm areas (~2% TBSA). |
| 10 | Age: 10 years | 150 | 45 | Left lower extremity | 3 | superficial_partial | Pink-red, moist areas around the distal leg and medial/plantar foot with epidermal peeling/blister edges. No waxy white, mottled, or leathery eschar; hair follicles remain visible—suggesting superficial partial-thickness. Estimated area: near-circumferential 8–10 cm band of distal leg (~25% of lower-leg surface) plus medial/plantar foot patch (~40% of foot), totaling about 3% TBSA. |
| 11 | Age: 2 years | 99 | 15 | Right lower extremity | 2 | superficial_partial | Right distal thigh/knee shows a pink, moist burn with shiny surface and partial epidermal loss; collapsed blisters/peeling at the margins. No leathery eschar or pale, mottled, insensate areas. These cues favor superficial partial-thickness. Involved area is limited to a small patch over the lateral distal thigh/knee, roughly ~2 child handprints, approximating about 2% TBSA. |
| 12 | Age: 1 years | 65 | 10 | Right upper extremity | 2 | superficial_partial | Distal forearm/wrist and radial palm show moist, bright pink/red dermis with ruptured blisters and sloughed epidermis; glossy surface suggests preserved perfusion. No waxy white, mottled, leathery, or eschar areas to suggest deeper injury. Involvement approximates ~1.5–2 infant palmar surfaces, giving about 2% TBSA from this region. |
| 13 | Age: 10 years | 135 | 40 | Right lower extremity | 3 | superficial_partial | Moist, pink glistening denuded areas with ruptured blisters along anterior knee–shin; peeling epidermis. No leathery or charred eschar, and no waxy mottled pallor, indicating preserved dermal perfusion. These cues favor superficial partial‑thickness. Surrounding diffuse erythema excluded. Involved area ≈ three child palms (~3% TBSA). |
| 14 | Age: 1 years 8 months | 65 | 15 | Left upper extremity | 3 | superficial_partial | Three discrete lesions on left upper arm and forearm show bright pink, moist wound beds with ruptured blisters and glossy surface; the child reacts painfully, indicating preserved sensation. No leathery/eschar or waxy white insensate areas. Combined area ≈ three child palms, about 3% of total body surface. |
| 15 | Age: 6 years | 120 | 20 | Face | 3 | superficial_partial | Pink moist areas with preserved texture, glistening surface, and no visible eschar or leathery/insensate zones indicate superficial partial-thickness. Distribution covers approximately one side of the face (≈3% TBSA for a 6-year-old). |
| 16 | Age: 6 years | 120 | 20 | Right upper extremity | 1 | superficial_partial | Cluster of moist, pink, glistening denuded patches with remnants of ruptured blisters in the right axilla. Uniform pink color suggests brisk capillary refill; no waxy/white, mottled, or leathery eschar to suggest deeper injury. Surrounding faint erythema is excluded. The total partial‑thickness area is smaller than the child’s palm (≈1% TBSA) and roughly half-palm in size, so rounded TBSA contribution is 1%. |
| 17 | Age: 1 years | 50 | 10 | Face | 9 | superficial_partial | Facial region shows moist, pink areas with preserved blanching and intact sensation; no leathery or insensate zones. These findings indicate superficial partial-thickness injury. The face in a 1-year-old approximates 9% of total body surface area. |
| 18 | Age: 1 years | 50 | 10 | Anterior thorax | 9 | superficial_partial | The anterior thorax shows moist, pink, peeling skin with preserved pain response and no leathery or insensate areas, consistent with superficial partial-thickness burns. In a 1-year-old, the anterior thorax represents about 9% of TBSA. |
| 19 | Age: 5 years | 110 | 22 | Right lower extremity and gluteal region | 7 | superficial_partial | The affected area involves the right buttock and posterior proximal thigh, roughly 7% TBSA for a 5-year-old. The wound is pink, moist, with visible blistering and no leathery or pale waxy zones. Capillary refill appears preserved and no insensate regions are visible, indicating superficial partial-thickness injury. |
| 20 | Age: 1 years | 60 | 13 | Face | 2 | superficial_partial | Shiny, moist pink-red area with serous exudate and small ruptured blisters across most of the forehead; no leathery eschar or white/mottled zones. These features indicate superficial partial-thickness. Area confined to forehead (~80% of forehead ≈ about one-third of face). For a 1-year-old, face ≈8–9% TBSA, giving ~2% TBSA involved. |
| 21 | Age: 6 years | 110 | 25 | Left upper extremity | 4 | superficial_partial | The left upper extremity shows pink, moist, shiny areas with some blistering and uniform color, consistent with superficial partial-thickness burn. Capillary refill appears intact and no leathery, pale, or insensate zones are visible. In a 6‑year‑old, the entire arm represents about 9% TBSA; only part is involved, estimating ~4% partial-thickness burn. |
| 22 | Age: 3 years | 112 | 13 | Left lower extremity | 2 | superficial_partial | The burn area is moist, pink-red, with visible sloughing of the epidermis and no leathery or pale insensate zones. Brisk capillary refill and preserved pain suggest superficial partial-thickness injury. The affected zone involves roughly the anterior aspect of the left lower leg only, representing about 2% TBSA in a 3-year-old. |
| 23 | Age: 15 years | 182 | 61 | Right lower extremity | 3 | superficial_partial | The burn involves the distal right leg and dorsum of the foot, roughly 3% TBSA. The wound shows moist, shiny pink areas with preserved hair follicles and no leathery or mottled zones, consistent with brisk refill and pain, indicating superficial partial-thickness injury. |
| 24 | Age: 16 years | 197 | 100 | Right upper extremity | 1 | superficial_partial | The burn involves the distal right forearm and dorsal hand, showing pink moist surface, preserved pain, and brisk capillary refill without leathery or insensate areas. Only a small localized area (<1% TBSA) is partial-thickness; the rest shows erythema only. These characteristics indicate a superficial partial-thickness burn. |
| 25 | Age: 0.5 year | 50 | 8 | Thorax | 12 | superficial_partial | The thoracic burn area appears bright red, moist, and uniformly pinkish without leathery or pale areas; this suggests intact capillary refill and surface moisture typical of superficial partial-thickness burns. Estimated thoracic involvement covers roughly one side of the anterior torso in an infant, about 12% TBSA. |
| 26 | Age: 0.5 year | 50 | 8 | Left lower extremity | 14 | superficial_partial | The left lower extremity shows bright red, moist, glossy burns with preserved contour and no leathery or pale areas. These features indicate superficial partial-thickness injury. In infants, the entire lower limb corresponds to roughly 14% TBSA; affected area appears to involve nearly all of the left leg. |
| 27 | Age: 3 years | 100 | 17 | Left lower extremity | 2 | superficial_partial | Anterior left shin shows a well-demarcated pink, moist patch without leathery texture, eschar, or pale/insensate areas; suggests good perfusion consistent with superficial partial-thickness. No charring or waxy white areas. Approximate size equals about two child palm areas, giving ~2% TBSA for this region. Surrounding erythema excluded. |
| 28 | Age: 16 years | 170 | 64 | Left lower extremity | 2 | deep_partial | Approximate area equals about two patient palms on the dorsum foot and medial ankle. Most skin is moist, pink, and blistered/peeling—consistent with superficial partial thickness. A focal medial malleolar area looks pale/waxy with yellow slough and reduced blanching, suggesting deeper dermal involvement. No leathery, charred, or insensate eschar to indicate full thickness. |
| 29 | Age: 2 years | 90 | 11 | Left lower extremity | 3 | superficial_partial | Moist, pink surface with preserved blanching and no leathery or white areas suggest superficial partial-thickness burn. In a 2-year-old, the left lower limb (leg + foot) corresponds to about 14% TBSA; only distal anterior portion is affected, involving roughly one-fifth of the region (~3% TBSA). |
| 30 | Age: 7 years | 110 | 28 | Right lower extremity | 3 | superficial_partial | Irregular moist pink-red denuded areas over the anterior ankle and dorsum of foot with peeled blister roofs. No leathery/charred eschar, and no extensive mottled white or waxy zones suggesting deep injury. Surrounding erythema excluded. Size approximates about three child-palms (~3% TBSA). |
| 31 | Age: 5 years | 110 | 15 | Left lower extremity | 1 | superficial_partial | The burn involves a small patch on the distal left leg and dorsal foot, representing about 1% of body surface in a 5-year-old. The wound bed is pink, moist, with visible capillary refill and no leathery or pale areas, consistent with superficial partial-thickness injury. |
| 32 | Age: 8 years | 110 | 20 | Left lower extremity | 2 | superficial_partial | Anterior–lateral knee/proximal leg shows moist, bright pink dermis with ruptured blisters and slough, indicating viable perfusion. No leathery, white, or charred areas suggesting full thickness. Predominantly uniform pink rather than mottled pale/white of deep partial. Involved area approximates two child palm sizes (~2% TBSA). Surrounding faint erythema excluded from calculation. |
| 33 | Age: 1 years | 80 | 10 | Right lower extremity | 1 | superficial_partial | Burn limited to plantar–medial right foot/heel, about the size of one pediatric palm (~1% TBSA). Pink, moist dermis exposed with large ruptured/intact blisters and glossy surface indicating preserved perfusion. No leathery eschar, charring, or waxy/mottled pallor—features favor superficial partial‑thickness over deeper injury. |
| 34 | Age: 4 years | 110 | 14 | Right upper extremity | 3 | deep_partial | Burn involves nearly entire hand (palmar and dorsal) plus a small distal forearm cuff, approximating three patient palms (~3% TBSA). Wounds are moist and red with de-roofed blisters; dorsal hand shows mottled pale/whitish areas and deep red tones, consistent with deeper dermal injury. No leathery black eschar or thrombosed vessels, arguing against full-thickness. |
| 35 | Age: 2 years | 90 | 14 | Left lower extremity | 2 | superficial_partial | The burn shows moist, pink-red areas with some blistering, indicating viable dermis and intact capillary refill. No signs of waxy, leathery, or insensate tissue. The region involved (anterior-lateral thigh in a toddler) represents about 2% of total body surface area. |
| 36 | Age: 61 years | 176 | 93 | Left upper extremity | 3 | superficial_partial | Distal forearm shows confluent bright pink-red, moist surface with ruptured blistering/denudation—features of superficial partial-thickness. Hair follicles remain visible and there is no leathery, white, or charred eschar. A small elbow patch appears deeper but limited. Involved area approximates 2–3 patient palms (circumferential band of distal forearm plus a small elbow patch), estimating about 3% TBSA for this region. |
| 37 | Age: 32 years | 150 | 60 | Left upper extremity | 1 | superficial_partial | Localized blistering with moist pink dermis, good capillary refill and tenderness indicate a superficial partial‑thickness burn. Involves a small patch on the distal forearm, roughly 1% TBSA for the left upper extremity region excluding uninjured areas. |
| 38 | Age: 30 years | 162 | 53 | Left lower extremity | 1 | superficial_partial | Moist, pink-red wound on dorsum of left foot with shiny surface and some ruptured blisters; no leathery/charred eschar or waxy pallor. Findings indicate viable dermis consistent with superficial partial-thickness. Affected area is roughly around one patient palm in size, excluding surrounding erythema, about 1% TBSA. |
| 39 | Age: 20 years | 169 | 59 | Left lower extremity | 1 | superficial_partial | Left foot dorsum and toes show multiple clear, tense blisters with pink, moist underlying skin. No leathery/eschar or waxy white areas; surrounding skin appears viable with visible hair proximally. These features indicate superficial partial-thickness injury. Involvement limited to dorsal foot/toes only, approximating one palm area (~1% TBSA). |
| 40 | Age: 19 years | 175 | 63 | Right lower extremity | 2 | superficial_partial | Moist, bright red, glistening wound with peeled blister edges on the medial ankle–foot. No leathery/charred eschar or pale, mottled, or waxy areas. Appearance suggests preserved dermal perfusion (surrogate for brisk capillary refill). These cues indicate superficial partial-thickness injury. Area equals roughly two patient palms over the medial ankle and arch, approximating 2% TBSA. |
| 41 | Age: 30 years | 168 | 77 | Right upper extremity | 1 | superficial_partial | Small, localized dorsal hand involvement with clear, tense blisters over ring finger and dorsum. Underlying skin appears pink and moist without waxy pallor, mottling, or leathery eschar—consistent with superficial partial thickness and preserved capillary refill. Burned area is a small fraction of the patient’s palm size (<1% TBSA); surrounding erythema excluded from count. |
| 42 | Age: 37 years | 170 | 86 | Left lower extremity | 4 | superficial_partial | The burn involves the anterior and lateral surfaces of the left lower leg and dorsum of the foot, roughly 4% TBSA by rule of nines for an adult. The area appears pink-red, moist, with intact or ruptured blisters and good capillary refill—consistent with a superficial partial-thickness burn. |
| 43 | Age: 37 years | 170 | 86 | Face | 1 | superficial_partial | Patchy, moist pink denuded areas on forehead, nose, and both cheeks with ruptured blisters/peeling. Surfaces appear shiny and hyperemic, implying brisk capillary refill. No waxy white, mottled, or leathery eschar; follicular dots intact. Involvement is limited to scattered portions of the face, totaling roughly a small fraction of the facial surface (<1–2% TBSA of the whole body). |
| 44 | Age: 37 years | 170 | 86 | Right lower extremity | 5 | superficial_partial | Burn involves most of the right lower leg from below knee to ankle, sparing the foot—about three‑quarters of the circumference and ~90% of length (~5% TBSA). Skin is pink-red and moist with multiple tense clear blisters; shiny surface suggests preserved perfusion. Hair follicles are visible. No waxy white, fixed maroon, or leathery eschar. Overall consistent with superficial partial-thickness injury. |
| 45 | Age: 24 years | 175 | 80 | Right lower extremity | 3 | superficial_partial | Large intact and ruptured blisters over foot/ankle with moist, bright-red dermis exposed; no leathery eschar or waxy white areas. Capillary refill surrogates: glistening, hyperemic surface. Hair follicles visible at margins. Burn involves most of the foot with a small circumferential ankle patch, approximating 3% of total body surface. |
| 46 | Age: 24 years | 175 | 80 | Anterior thorax | 7 | superficial_partial | Large, tense blisters with clear fluid over most of both breasts and upper chest; underlying dermis appears pink and moist. No leathery/charred eschar or pale mottled areas. These cues indicate superficial partial-thickness. Approximately 70–80% of the anterior chest (≈9% TBSA) is involved, giving ~7% TBSA for this region. |
| 47 | Age: 39 years | 165 | 95 | Right upper extremity | 4 | superficial_partial | The burn involves the posterolateral right upper extremity with moist, pink areas, intact pain response, and no leathery or insensate zones. Capillary refill appears brisk and no eschar is visible, indicating a superficial partial-thickness injury. The right upper extremity (shoulder to wrist) represents about 9% TBSA; the affected portion is less than half, approximating 4%. |
| 48 | Age: 39 years | 165 | 95 | Left upper extremity | 1 | deep_partial | Lesions over dorsal fingers with mixed pale and dark mottled areas, crusted surface, and poor visible capillary refill indicate deep partial-thickness injury. The remainder of the hand and arm appears intact. The involved dorsal finger zones represent about 1% of total body surface area. |
| 49 | Age: 57 years | 155 | 65 | Right upper extremity | 9 | superficial_partial | The right upper extremity from shoulder to wrist shows a moist, pink, blanching surface with preserved skin texture and no leathery eschar or pallor. These features indicate superficial partial-thickness injury. For an adult female, the entire right upper extremity represents about 9% TBSA. |
| 50 | Age: 57 years | 155 | 65 | Anterior thorax | 9 | superficial_partial | The anterior thorax shows bright erythema with some moist pink areas and minimal blistering. Capillary refill appears preserved and there are no waxy, mottled, or insensate zones, consistent with superficial partial-thickness injury. The anterior trunk represents about 9% of total body surface area in an adult female. |
| 51 | Age: 30 years | 165 | 80 | Left lower extremity | 1 | superficial_partial | Cluster of thin-walled, clear blisters over medial/dorsal left ankle/foot with moist, pink underlying dermis and surrounding blanching erythema. No pale/mottled insensate areas, no leathery eschar or charring; hair follicles visible proximally—features consistent with superficial partial-thickness. The blistered/denuded area is roughly half to one patient palm in size; excluding surrounding erythema, this equals about 1% of total body surface. |
| 52 | Age: 26 years | 176 | 52 | Right lower extremity | 2 | superficial_partial | Burn involves the dorsum of the right foot extending to toes and a small patch at the anterior ankle. Wound bed is bright red, moist, and glistening with sloughed/ruptured blisters; hair follicles are visible; no leathery, white, or charred eschar. These cues indicate superficial partial-thickness. Area roughly equals about two patient hand areas (1% each), excluding surrounding erythema, giving ~2% TBSA. |
| 53 | Age: 47 years | 162 | 62 | Face | 4 | superficial_partial | Facial skin shows moist pink areas with intact adnexal structures and preserved hair follicles, no leathery eschar or waxy mottling, and likely preserved sensation—consistent with superficial partial-thickness burns. The face represents approximately 4% of total body surface area in adults. |
| 54 | Age: 47 years | 162 | 62 | Left upper extremity | 1 | superficial_partial | Multiple small patches on the left forearm show moist, pink dermis with clear/ruptured blisters and visible hair follicles; no leathery or insensate white/black eschar. These features indicate superficial partial-thickness injury. The summed denuded areas are less than a patient’s palm (~1% TBSA); surrounding erythema without skin loss was excluded from the estimate. |
| 55 | Age: 34 years | 183 | 90 | Left upper extremity | 1 | deep_partial | Single oval burn on dorsum of left hand, well under the patient’s palm size (~1% TBSA), so 1%. Wound bed shows central pale/cream moist slough with scattered punctate bleeding; peripheral bright pink rim and collapsed blister edge. Mottled color with reduced blanching suggests deeper dermal injury; no leathery/charred eschar, favoring deep partial thickness. |
| 56 | Age: 53 years | 160 | 68 | Left lower extremity | 3 | deep_partial | The left lower limb shows irregular, mixed red-yellow areas with adherent eschar and dull capillary response over the shin and posterior knee. Texture appears dry and mottled rather than moist, suggesting deep partial-thickness injury. The affected surface, limited to small patches on the leg and knee of one limb, represents roughly 3% TBSA. |
| 57 | Age: 21 years | 170 | 62 | Left upper extremity | 1 | superficial_partial | Small intact clear-yellow blister on the thenar eminence with pink, moist surrounding skin and preserved palmar lines; no leathery eschar, charring, or insensate white areas. Rest of left upper extremity appears normal. Involved area is only a few cm on the palm—well under the palmar 1% TBSA; reported as 1% due to integer requirement. |
| 58 | Age: 18 years | 155 | 55 | Left upper extremity | 1 | superficial_partial | Dorsoradial hand shows an unroofed oval blister with a moist, pink wound bed and adjacent intact blister. Surrounding erythema is excluded. No waxy white, mottled, or leathery/eschar areas. These features support superficial partial-thickness. Involved area is much smaller than the patient’s palm (~1% TBSA), roughly one‑third of a palm; rounded to 1%. |
| 59 | Age: 35 years | 180 | 101 | Left upper extremity | 1 | superficial_partial | Moist, pink wound on dorsal hand with de-roofed blister and shiny surface indicates viable dermis and capillary refill; skin lines/hair follicles remain visible. No leathery, white/charred eschar or dry, insensate patches to suggest deeper injury. Burned area is a small patch on the dorsum/wrist, well under the area of one patient palm (~1% TBSA); surrounding erythema/ecchymosis not counted. |
| 60 | Age: 19 years | 178 | 65 | Thorax | 3 | superficial_partial | Irregular bright‑pink, moist, glistening areas with epidermal loss over left upper chest/shoulder. Hair follicle dots are visible; no leathery, charred, or mottled white patches. These cues indicate preserved perfusion and superficial partial‑thickness injury. Area approximates ~3 patient palms (~3% TBSA). Surrounding simple erythema is excluded from the estimate. |
| 61 | Age: 24 years | 160 | 60 | Right lower extremity | 1 | superficial_partial | Blistering localized to dorsal right foot; intact and ruptured clear bullae over a pink, moist, shiny dermis that appears to blanch. No leathery/eschar or waxy white areas. Counting only the blistered portion, the area is about one patient palm, ≈1% of total body surface. |
| 62 | Age: 42 years | 170 | 85 | Face | 1 | superficial_partial | Localized pink, moist facial burn with intact surface gloss and no leathery or pale areas. Capillary refill appears preserved, suggesting viable dermis and superficial partial-thickness injury. The affected area is limited to a small patch on one cheek, approximately 1% of total body surface area. |
| 63 | Age: 42 years | 170 | 85 | Left upper extremity | 4 | superficial_partial | Left upper extremity shows multiple pink, moist, weeping areas with ruptured blisters on upper arm and forearm. These features indicate viable dermis and capillary refill, consistent with superficial partial thickness. No leathery, white, or charred eschar to suggest deeper injury. Summed involved area equals roughly 3–4 patient palms, excluding surrounding erythema, giving about 4% TBSA. |
| 64 | Age: 42 years | 170 | 85 | Right lower extremity | 2 | superficial_partial | Right lower leg shows multiple tense, clear blisters over pink, moist skin; hair follicles visible and no leathery or white eschar—features of superficial partial-thickness. Burned areas are patchy linear strips along anterior–lateral shin with small spots on distal thigh/ankle; surrounding erythema excluded. Overall area is a small fraction of the limb, approximating ~2% of total body surface. |

**Supplementary Table S1. Case-level LLM outputs for burn region-cases.** This de-identified dataset contains one row per burn region-case (n = 64) and includes the following columns: case_no (anonymous region-case identifier), age_text (standardized English age information), height_cm, weight_kg, region (anatomical site in English), tbsa_percent (LLM-estimated percentage-point contribution of the region to total body surface area), depth_class (LLM depth classification: superficial_partial, deep_partial, or full_thickness), and rationale (≤80-word free-text explanation generated by the LLM).
