# Supplementary material for: Multimodal large language model versus emergency physicians for burn assessment: a prospective non-inferiority study
Source: Scand J Trauma Resusc Emerg Med. 2026 Feb 5;34:54. doi: 10.1186/s13049-026-01577-6 (PMC12969848; doi:10.1186/s13049-026-01577-6)
Supplement: Supplementary file 3 — Supplementary Material 3. [file 13049_2026_1577_MOESM3_ESM.docx]

| **Panel / LLM** | **superficial_partial** | **deep_partial** | **full_thickness** | **Total** |
| --- | --- | --- | --- | --- |
| superficial_partial | 45 | 2 | 0 | 47 |
| deep_partial | 13 | 3 | 0 | 16 |
| full_thickness | 1 | 0 | 0 | 1 |
| **Total** | 59 | 5 | 0 | 64 |

| **Panel / Physicians** | **superficial_partial** | **deep_partial** | **full_thickness** | **Total** |
| --- | --- | --- | --- | --- |
| superficial_partial | 40 | 6 | 1 | 47 |
| deep_partial | 2 | 13 | 1 | 16 |
| full_thickness | 0 | 0 | 1 | 1 |
| **Total** | 42 | 19 | 3 | 64 |

| **Physicians / LLM** | **superficial_partial** | **deep_partial** | **full_thickness** | **Total** |
| --- | --- | --- | --- | --- |
| superficial_partial | 40 | 2 | 0 | 42 |
| deep_partial | 17 | 2 | 0 | 19 |
| full_thickness | 2 | 1 | 0 | 3 |
| **Total** | 59 | 5 | 0 | 64 |

**Supplementary Table S2. Confusion matrices for burn depth classification.**

(A) LLM versus expert panel. (B) Physician consensus versus expert panel. (C) LLM versus physician consensus.

Rows represent reference categories and columns predicted depth classes (superficial_partial, deep_partial, full_thickness); cell values are the number of burn region-cases in each category combination.
